# Human exposure to uranium in South African gold mining areas using barber-based hair sampling

**DOI:** 10.1371/journal.pone.0219059

**Published:** 2019-06-27

**Authors:** Frank Winde, Gerhard Geipel, Carolina Espina, Joachim Schüz

**Affiliations:** 1 North-West University, Vaal Triangle Campus, Research Unit Environmental Science and Management, Mine Water Re-Search Group, Vanderbijlpark, South Africa; 2 Helmholtz-Zentrum Dresden-Rossendorf, Institute for Resource Ecology, Dresden, Germany; 3 International Agency for Research on Cancer (IARC), Section of Environment and Radiation, Lyon, France; University of Witwatersrand/NHLS, SOUTH AFRICA

## Abstract

Uranium (U) measurements in water, soil, and food related to gold mining activities in populated areas in Gauteng Province, South Africa, suggest the possibility of exposure levels that may lead to adverse health consequences, including cancer. Theoretical considerations on pathways of human uptake of significant exposures are plausible, but few data on directly measured human exposure are available. A cross-sectional study was conducted using human measurements to compare U levels with other settings around the globe (based on literature review), to explore potential exposure variability within the province, and to test the feasibility of recruiting subjects partially coming from vulnerable and difficult-to-reach populations. Wards of potentially high (HE) and low exposure (LE) were identified. Composite hair samples representing the respective local populations were collected from regular customers of selected barber shops over a period of 1–2 months. A total of 70 U concentrations were determined in 27 composite samples from 1332 individuals. U concentrations ranged from 31 μg/kg to 2524 μg/kg, with an arithmetic mean of 192 μg/kg (standard deviation, 310 μg/kg) and a median of 122 μg/kg. Although HE wards collectively showed higher U levels than LE wards (184 vs 134 μg/kg), differences were smaller than expected. In conclusion, detected U levels were higher than those from most other surveys of the general public. The barber-based approach was an efficient hair collection approach. Composite hair samples are not recommended, due to technical challenges in measuring U, and individual hair samples are needed in follow-up studies to determine predictors of exposure.

## Introduction

At many South African gold mines, the ore extracted contains not only gold but also considerable amounts of uranium (U), which is brought to the surface inadvertently. Mine waste in hydraulically generated deposits (locally called “tailings dams”) cover about 400 km^2^, mostly in regions that, through mining, developed into densely populated urban agglomerations, like Johannesburg. The gold tailings of the Witwatersrand basin have an average U concentration of about 100 mg/kg U_3_O_8_ (ranging from about 10 mg/kg to several hundreds of mg/kg); these gold tailings thus contain as much U as, or more U than, tailings from dedicated U mines, for example those in Germany or Namibia [1]. It has been estimated that in Gauteng Province alone, approximately 1.6 million people live in close proximity to tailings dams [2], and growing informal settlements are moving closer and closer to them [3]. The pathways through which local residents are exposed include inhalation of windblown tailings dust and radon; consumption of polluted river water and groundwater; ingestion of food produced with contaminated water, including home-grown vegetables, meat and milk from domestic livestock, and fish from polluted water; and intentional (geophagia) and unintentional (hand-to-mouth) consumption of contaminated soil, sediment, and tailings material [1, 4–6]. From a review of environmental U measurement surveys in the area and predictions of people’s exposure, performed during an expert workshop held in Johannesburg in 2013, it was concluded that direct measurements in human populations living around the tailings were needed to assess exposure distributions and conditions [7]. The aim of such measurements would be to confirm that theoretical considerations on the multiple exposure pathways and high environmental levels were indeed resulting in an increased exposure in humans.

Health effects reported for U are attributed to the heavy metal chemotoxicity and, to a lesser extent, to its radioactive properties (e.g. [8]), which are of growing concern if U is inhaled or ingested. Alpha-emitters in general have been classified as carcinogenic to humans by the IARC Monographs programme [9]. The classification was based on mechanistic considerations insofar as alpha-emitters for which there was less convincing direct human evidence were not expected to differ in their carcinogenic potential at the same doses compared with alpha-emitters for which there was established direct human evidence, such as radium (-224, -226, -228) or plutonium-239. From the epidemiological studies on the carcinogenicity of mixtures of U isotopes, the conclusion was that there was only limited evidence in humans, in line with the previous IARC assessment [10]. Although in some studies the mortality rates from lung cancer and other site-specific cancers were found to increase, clear interpretation was hampered by lack of consistency, the difficulty in measuring the dose of radiation, potential concomitant exposure to chemicals, and possible healthy worker effects [10]. However, given the chemotoxicity and radioactive properties, the concerns about the carcinogenicity of U remain.

Recent findings from investigations of health effects of U, including depleted U used in military conflicts, suggest a much wider range of toxicity, including teratogenicity [11–13], disruption of the endocrine system by U mimicking estrogen’s effects [14], and genetic damage [15–17] as well as neurotoxic effects [18–20]. Therefore, if high U levels were confirmed in human populations exposed to the tailings, investigations of subsequent adverse health effects, including cancer, would be justified.

To have a first overview of the distribution of human exposure in the potentially affected populations, a pilot study was designed to include diverse populations, ranging from middle-class and less-affluent neighbourhoods to the most vulnerable populations in informal settlements, and including all races. This required non-invasive, ad hoc collection of biological specimens by non-specialists, to deliver reliable measurements of U concentrations, and an easy approach to reach a large number of subjects without verbose explanations on-site. Hair was considered to be an appropriate specimen, given its use in previous studies [21–28]. In addition, a literature review was performed to compare the U concentrations in the study area with those in other settings that applied comparable methods to measure U concentrations in humans.

Therefore, the present study had three objectives: (1) to test whether the sample measurements in human populations confirm the expected higher U levels suggested from environmental measurements and theoretical considerations of plausible uptake pathways; (2) to investigate whether there are exposure differences, by sampling from neighbourhoods of presumably high or low exposure intensity as a result of their location in relation to the mine tailings and other mining-related U sources; and (3) to find out how well the study protocol performs and what would need to be optimized for any larger-scale longitudinal study.

## Materials and methods

### Study population

Wards (a ward is the smallest administrative unit of population counting in South Africa) were identified according to expected exposure likelihood and racial diversity, given the different living conditions of White, Indian, and Black populations that still largely prevail in post-apartheid South Africa. The expected probability of exposure was assessed based on the proximity of settlements to mine tailings as well as the anticipated degree of environmental U pollution (rivers, mine water canals, soil including tailings, and dust) as known from previous environmental measurement studies. Exposure pathways considered in this context included drinking polluted water (e.g. untreated river water), ingesting contaminated food (including fish from polluted rivers and vegetables, crops, and meat and milk livestock produced with contaminated water and soil), direct consumption of tailings and polluted soil or sediment (geophagia as well as unintended hand-to-mouth transfer, mainly in children), swallowing polluted water while swimming, and inhalation of windblown tailings dust.

With the aim of selecting worst-exposure scenarios, nine wards of high exposure (HE) probability were selected, representing the locally dominant Black, White, and Indian populations and, to a lesser extent, also Coloured (mixed-race) populations. Six of those wards were located in the West Rand goldfield (Tudor Shaft, Kagiso Extension 6, Kagiso Extension 8, Rietvallei (all Black), Azaadville (Indian) and Mindalore (White)), most of them in close proximity to the upper Wonderfonteinspruit, a heavily mining-polluted stream [1]. Khutsong North (Black), a township of the Far West Rand goldfield further downstream of the Wonderfonteinspruit stream was also selected, as well as two wards in the western part of the Central Rand goldfield (Diepkloof Zone 4 (Black) and Noordgesig (Coloured)). For each Black, Indian, and White population, a corresponding ward of low exposure (LE) probability was selected, aiming for similarity in socioeconomic living conditions and a location well outside the various goldfields, namely Alexandra (Black), Laudium (Indian), and Randburg (White) (none identified for Coloured). These 12 wards, shown in S1 Fig, form the study population.

Aided by a local nongovernmental organization with close links to the communities, we identified barber shops in each ward serving local customers of both sexes (except in Azaadville, where the chosen barber turned out to serve only men). The barbers, who generally enjoy a trust-based relationship with long-term clients, were instrumental in explaining the aim of the study to their customers and addressing concerns about potential misuse of the hair as “muti” (traditional medicine). The barbers confirmed in a questionnaire that most of their clients came from a radius of approximately 2 km around the barber shop, thus representing the local population. Samples were compiled from the customers visiting the barber during the sampling period (1–2 months). Efforts were made to identify low-cost barbers, in order to include indigent groups such as informal settlers, who were expected to be among the most exposed.

Barbers collected hair from up to 100 individuals per sex, following a detailed sampling protocol and instructions stipulating, inter alia, that they should collect a sufficient amount of hair per individual (at least 1 cm in length). Samples were totally anonymized. The instructions included not collecting dyed or otherwise chemically treated hair or artificial hair extensions, or hair that had fallen onto the floor, and to obtain oral consent from customers. Composite samples of the same weight of hair were created by barber and sex and shipped to a laboratory in Germany for the U analyses.

The study was approved by the IARC Ethics Committee. Customers were informed by the barber about the purpose of the study, but because no other information was recorded apart from sex, age group (minor, adult, or elderly), and race, the institutional review board (IARC Ethics Committee) approved the use of oral consent (documented by the barber confirming that only hair samples of customers giving oral consent were provided).

#### Additional samples

In addition to the samples from the study population, we obtained further samples that were not part of the study population. Results for these additional samples are reported here because those were also informative regarding the bigger picture of U exposure in the region. In Noordgesig, some residents were approached directly by field assistants, including a family of four suffering from an unspecified sickness. In addition, the barber in Mindalore provided two bags, containing hair from 9 boys and 2 girls, respectively, which were treated as special composite samples. For age comparison, samples of fully grey hair were collected from Mindalore characterizing elderly clients (25 male, 6 female). Other special individual samples included hair from a White boy who relocated from Potchefstroom (South Africa) to Germany, as well as a very long cut of hair from a Black woman from Kagiso. The locations where these special samples were obtained are also included in S1 Fig.

### Uranium measurement

Based on recommendations by Karpas et al. [25], the homogenized hair was rinsed using distilled water, a surfactant (Triton X-100), and ethanol (or acetone) combined with sonication, which was intended to remove external contaminants, such as tailings dust. To dissolve the hair, a mixture of 1 ml of 70% nitric acid and 2 ml of 30% hydrogen peroxide was added, and this was either left overnight or expedited by putting the tube into a hot water bath at 80°C for 10 minutes. Before it was analysed, the sample in the test tube was diluted with distilled water to 15 ml. In addition, 10 μg of a certified iridium or bismuth standard was added to each sample as well as to blanks (digestive solutions containing no hair) to correct for the matrix effect and for fluctuations of the Inductively coupled plasma (ICP) torch, by adding an element that was not in the sample but was similar in mass to the element of interest. Comparison of the ICP-MS counts for iridium or bismuth between blanks and digested hair solutions indicated to what extent the matrix of the hair solution may have attenuated the signal. All parameters of the ICP-MS were set to optimal levels for U measurements, and the device was calibrated using certified standard solutions for U. The U concentrations measured in the digestive solutions were subsequently corrected for the matrix effect. Each sample was measured at least twice (duplicates). When the amount of hair was sufficient to allow further measurements or when the first two measurements differed by more than 50%, a third and fourth analysis was performed. Each measured sample was drawn individually from the composite sample bags. The U content in the hair sample (in μg/kg) was calculated by multiplying the corrected U concentration in the digestive solution (in μg/l) by 15,000 (to account for the 15 ml of added acid and water) and dividing this by the weight of the dried hair (before being digested) in mg.

### Statistical methods

Statistical methods include univariate statistics and their visualization in figures. Sex-weighted averages (arithmetic means for men and women weighted according to the number of men and women in the respective sample) were calculated to compare different wards when the sexes were combined, to take into account potential differences between the hair from men or from women (e.g. in terms of hair length and hair care). Acknowledging the small sample size of 70 measurements, we performed an analysis of variance (ANOVA) with the measured U levels as response variable and sex, race, and HE vs LE as explanatory variables mutually adjusted for each other, using a fixed-effect model (with SAS 9.4).

## Results

In total, 27 composite samples were collected from the 12 wards of the study population. These included 6 samples from LE wards, as planned, namely from 3 barbers serving both sexes. From HE wards, 21 samples were provided instead of the planned 18, for the following reasons. In the Kagiso Extension 8 and Rietvallei wards, hair collection was performed in two phases. As a result of concerns among local customers about the use of their hair, the fieldwork was interrupted and it was then decided not to mix the hair from the two phases (therefore, 8 samples were obtained from the two wards, instead of 4). In Azaadville, the selected barber turned out to serve only men (therefore, only 1 sample set was obtained, instead of 2). From the 27 composite samples (21 HE and 6 LE), 70 measurements of U were retrieved (see the Materials and Methods section). Overall, hair from 1332 individuals was analysed (Table 1).

**Table 1:** Uranium concentration in human scalp hair of 70 measurements in 27 composite hair samples of 1332 individuals from 12 selected wards in Gauteng Province, South Africa.

Exposure level	Ward	Race	Sex	*N*	U concentrations (μg/kg)				*N*	Mean	Sex-weighted mean^b^
				Subjects	1st run	2nd run	3rd run	4th run	Measurements	μg/kg	μg/kg
HE	Kagiso Extension 6	Black	Male	31	274	470	63	66	4	218	
			Female	81	121	192	108	109	4	133	
			*Combined*	112					8	175	156
HE	Kagiso Extension 8^a^	Black	Male (1^st^ phase)	49	73	57			2	65	
			Male (2^nd^ phase)	44	136	83	58	107	4	96	
			Female (1^st^ phase)	39	108	341	77	72	4	150	
			Female (2^nd^ phase)	40	2524	132	171	71	4	725	
			*Combined*	172					14	286	247
HE	Rietvallei^a^	Black	Male (1^st^ phase	50	125	125			2	125	
			Male (2^nd^ phase)	50	301	295			2	298	
			Female (1^st^ phase)	50	123	129			2	126	
			Female (2^nd^ phase)	48	191	193			2	192	
			*Combined*	198					8	185	186
HE	Diepkloof	Black	Male	33	222	156	116	124	4	155	
			Female	66	177	73	169	217	4	159	
			*Combined*	99					8	157	158
HE	Tudor Shaft	Black	Male	31	275	248			2	262	
			Female	20	87	109			2	98	
			*Combined*	51					4	180	197
HE	Khutsong North	Black	Male	55	660	611			2	636	
			Female	64	478	459			2	469	
			*Combined*	119					4	552	546
HE	Azaadville	Indian	Male	100	45	44			2	45	
HE	Noordgesig	Coloured	Male	11	110	96			2	103	
			Female	51	102	102			2	102	
			*Combined*	62					4	103	102
HE	Mindalore	White	Male	100	43	31	41	34	4	37	
			Female	33	56	46			2	51	
			*Combined*	133					6	42	41
LE	Alexandra	Black	Male	14	187	233			2	210	
			Female	10	159	155			2	157	
			*Combined*	24					4	184	188
LE	Laudium	Indian	Male	49	102	118			2	115	
			Female	26	121	134			2	128	
			*Combined*	75					4	119	116
LE	Randburg	White	Male	107	72	57			2	65	
			Female	80	154	133			2	144	
			*Combined*	187					4	104	98

HE, high exposure; LE, low exposure.

^a^ In Kagiso Extension 8 and Rietvallei, sample collection was interrupted and therefore took place in two phases.

^b^ Weighted according to number of samples per sex.

Table 1 shows the 2–4 individual measurement values in μg/kg dry hair and the summary statistics by ward and sex (men, women, and combined), with arithmetic means and sex-weighted arithmetic means for the two sexes combined. U values ranged from 31 μg/kg to 2524 μg/kg, with an average (arithmetic mean) across all measurements of 192 μg/kg (standard deviation, 310 μg/kg) and a median of 122 μg/kg (1st quartile: 74 μg/kg; 3rd quartile: 190 μg/kg). Only one measurement exceeded 1000 μg/kg.

**Fig 1:**
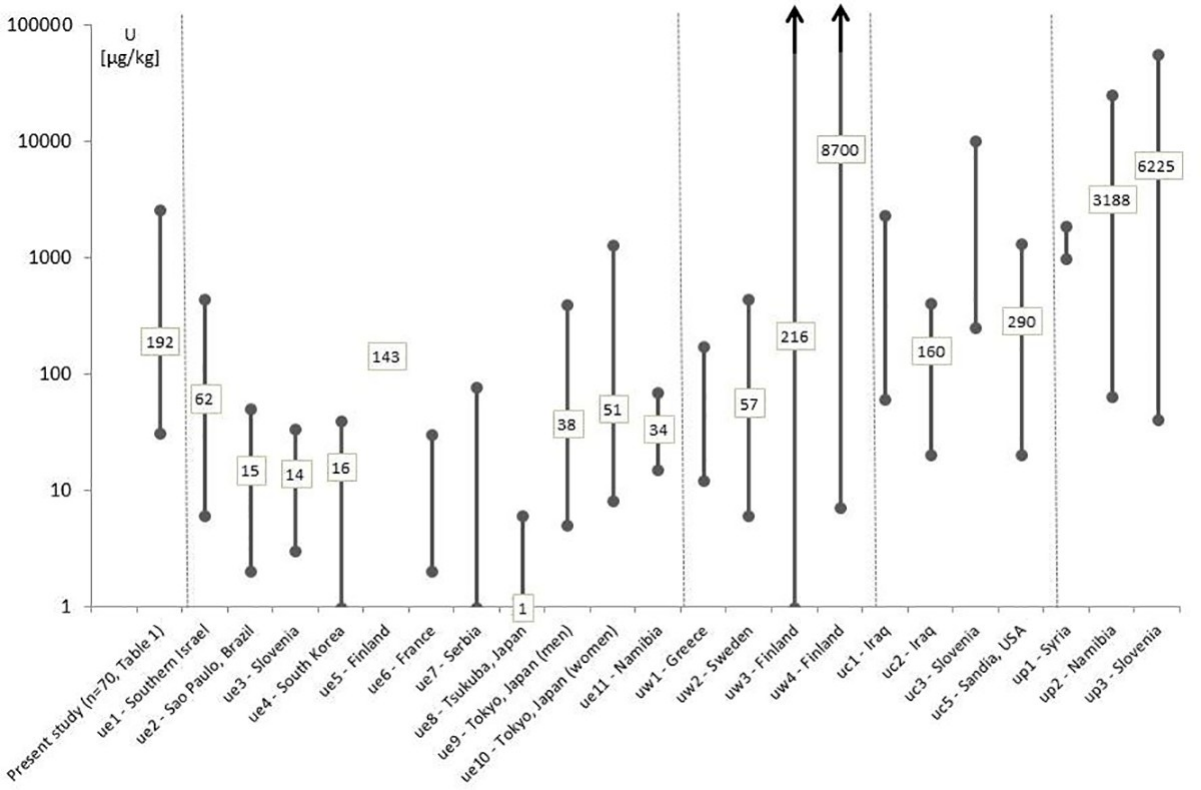
Uranium (U) concentrations in μg/kg measured in all composite hair samples and in high-exposure (HE) and low-exposure (LE) wards (as shown in Table 1) combined of the present study in Gauteng Province, South Africa, compared with international measurements of U concentrations in presumably unexposed populations (ue), populations with expected higher U in drinking water (uw), populations exposed to U contamination from nuclear facilities or depleted U in war zones (uc), or populations affected by mining or U production (up) (shown as means and ranges). Three-letter code in the study name indicates the study ID from S1 Table for reference.

To put those U concentrations into context, as stated in objective 1, Fig 1 contrasts our findings from the HE wards and LE wards (all those from Table 1) with results from other studies of U measurements in hair from different countries. These studies include surveys in supposedly unexposed populations and also in populations assumed to have increased U levels in hair due to occupational or environmental exposure. Among those, U concentrations reported by Byrne and Benedick [22] were particularly suited for comparison, because of their emphasis on data quality control. U levels in hair of workers and nearby residents of a U mine in Namibia as reported in Kudzu Science [29–30] are also of special relevance, because it is a southern African context, including ethnicity aspects, and links mining to impacts on nearby residents under similar natural conditions. The studies used are listed in S1 Table.

**Fig 2:**
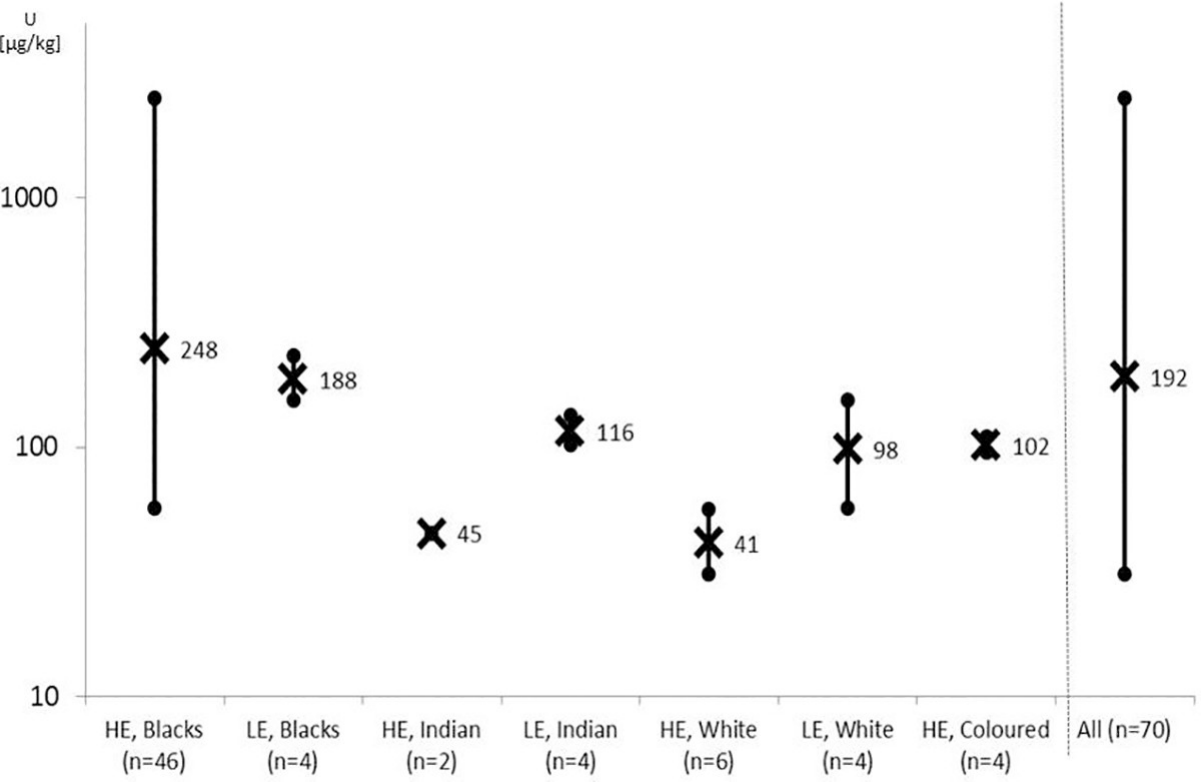
Uranium (U) concentrations in μg/kg measured in composite hair samples from assumed high-exposure (HE) and low-exposure (LE) wards by race (wards with mostly Black, Coloured, Indian, or White populations), with sex-weighted arithmetic mean (X) and range (maximum and minimum) in *n* measurements (*n* = number of composite samples measured). The “All” group comprises all 70 measured composite samples from HE and LE wards combined (all shown in Table 1).

Regarding objective 2, contrary to what was expected, the arithmetic means from LE White and Indian wards displayed higher instead of lower U concentrations than those of the corresponding HE White and Indian wards (98 vs 41 μg/kg, 110 vs 45 μg/kg (men only)). However, the LE Black ward showed lower levels than the sex-weighted average of all HE Black wards combined (188 vs 248 μg/kg). Collectively, U levels of HE wards were therefore not much higher than those of LE wards (186 vs 134 μg/kg). Fig 2 shows sex-weighted arithmetic means, maximum, and minimum for all wards by HE vs LE and race. Overall, higher U levels were measured in women than in men (140 vs 114 μg/kg), which also holds true for most ward–race combinations except for HE Coloured (equal) and LE Black (higher in men). ANOVA showed that these differences in arithmetic means are only suggestive and need to be confirmed with larger samples. In ANOVA, the effects of sex (*P* = 0.42), HE vs LE (*P* = 0.49), and race (*P* = 0.32) were not statistically significant, with an overall *R*^2^ of 7%.

Regarding objective 3, in addition to the insight gained into conducting such a study in a challenging setting, the most valuable information from Table 1 in this context is that variations within repeated measurements from the same composite sample were often substantial. This indicates that the mixing of samples from up to 100 individuals in a bag from which a composite sample for the U measurement is drawn does not lead to the same averaged U concentrations. Incomplete homogenization of mixed hair stems from effects such as electrostatic charging or lumping of curled hair, which even freezing and subsequent milling in a steel ball chamber could not overcome. Complete digestion of all mixed hair was impossible because of laboratory safety regulations limiting the volume of corrosive acids.

In addition, some information was derived from the special samples. Table 2 shows the 15 measurement values from the 5 special samples in μg/kg dry hair and the summary statistics by sample. Among these additional samples, U values ranged from 27 μg/kg to 3607 μg/kg, with an average (arithmetic mean) across all measurements of 636 μg/kg (standard deviation, 1194 μg/kg) and a median of 68 μg/kg. All three measurements of hair of 2 girls from Mindalore exceeded 1000 μg/kg. The latter group shows that exceptionally high values can be found. Notably, the measurements in the four sick people from Nordgeesig were not higher than the average from Table 1.

**Table 2:** Uranium concentration in human scalp hair of 15 measurements in 5 special samples from 46 individuals in Gauteng Province, South Africa.

Type of sample	Ward	Race	*Sex*	*N*	U concentrations (μg/kg)				*N*	Mean
				Subjects	1st run	2nd run	3rd run	4th run	Measurements	μg/kg
Special	Mindalore	White	Boys	9	145	115			2	130
			Girls	2	2875	2153	3607		3	2878
			Male (elderly)	25	52	52	38	68	4	53
			Female (elderly)	6	28	28	27	29	4	28
Special	Noordgesig	Coloured	Sick family	4	156	166			2	161

**Fig 3:**
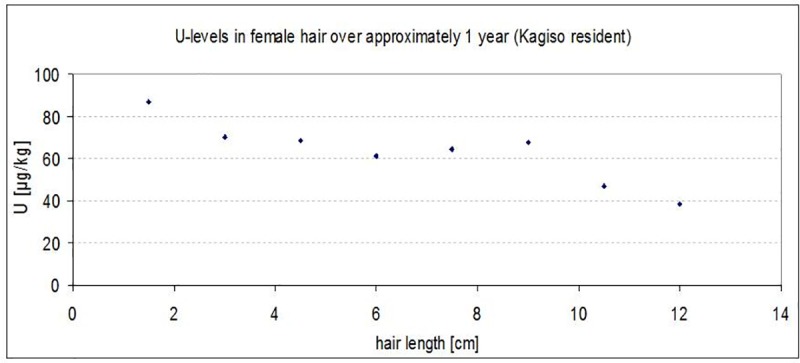
Uranium concentration measured at different lengths of 12 cm long hair from a Black woman from Kagiso.

Fig 3 shows U levels in hair from one individual with long hair, to examine U concentration in relation to the hair length at different time points. Notably, the concentration varies considerably over the 12 cm total length by a factor of more than 2. Even higher temporal variations were found in a White boy who relocated from South Africa to Germany, showing a decrease from 38 μg/kg while living in Potchefstroom, where the drinking water supply was affected by upstream U pollution [31], to 7 μg/kg after living for 10 months in Halle (Saale), with no elevated U concentrations in the drinking water.

## Discussion

Comparing the general population composite hair samples from our pilot study with uranium (U) concentrations reported in the literature for unexposed populations shows that the majority of our samples clearly exceeded background levels (objective 1). Notably, the arithmetic mean from our study population was higher than that among a population in Northern Sweden [32] and comparable to that of a population in Finland [24], both with increased U levels in their drinking water. Some of our measured values overlapped with measured values reported for workers in U and (uraniferous) phosphate mines in Slovenia, Namibia, and Syria. This confirms the rationale for conducting the pilot study, namely that the environmental levels above the normal background levels and plausible scenarios of human exposures do in fact lead to a measurably higher uptake. In addition, due to the lack of human data, risk assessments for Gauteng Province so far have relied only on modelling. However, inconsistencies in those assessments [33–37] support our approach, with measuring actual human exposure instead of modelling (original data for international comparison in S1 Table, extracted from references [13, 21–26, 29, 38–42]).

There was little difference in averages between the HE and LE wards and large overlaps in measured values, which was unexpected because the wards were chosen deliberately, mainly based on distance to tailings dams (objective 2). In fact, comparing male and female populations showed tendencies of consistently higher U levels for LE wards across all races and sexes except for Black females. There are three possible explanations for this finding. First, U in the environment may spread more widely than assumed; this is, however, purely speculative and is not supported by the available environmental measurements from previous surveys [7]. Second, individual habits may be more important than residence, and the individual choice of water and food, and also the occupation may lead to a dilution of measured group-level exposure. Third, and perhaps most likely, is the influence of the choice of barber shops. Re-contacting the barber shops at the end of study showed that many had more commuting customers than anticipated, and for any future study inclusion of the cheap street barbers in the HE wards is highly recommended to better capture residents from the local neighbourhood. Temporal variation appears to be another important factor, as illustrated in the, though only one, very long hair sample of 12 cm available to us. Although hair levels are supposed to represent a longer exposure period than, for example, urine levels, this variation casts doubt on how much biological samples can be used as predictors of exposure over several decades, as would be needed for investigating cancer risk.

Therefore, it remains an objective to develop an exposure prediction model based on residence, taking into account proximity to tailings dams, wind directions, erosion, water distribution and consumption, use of local food, and other potential exposure routes (including occupational), with the aim of looking at correlations between exposure and health outcomes on an ecological level, but the experience of this pilot study shows that validation of any such exposure maps would be essential before they could be applied.

Regarding objective 3, hair sampling through barber shops turned out to be an efficient and low-cost method of hair collection, including in the most deprived areas where it would be challenging, if not impossible, to collect other biological samples, such as blood (due to safety-assured medical staff needed for sampling) or urine (due to lack of even the most basic sanitary facilities). However, in these areas hair plays a major role in traditions and is suspected to be used in witchcraft, so barbers need to be carefully instructed on how to inform their customers, so as to not lose the trust of the local residents. We had expected that acceptance would be higher when explaining hair would be analysed for groups and not for the individual, but it did not matter as much as anticipated and acceptance turned out to be high for hair collection from individuals. Individual hair collection and U analysis should be foreseen for future studies because the potential benefit of ease of collection is outweighed by the fact that the treatment of composite hair in laboratory measurements is very difficult. Electrostatic charging and mechanical lumping of heavily curled hair prevents a balanced mixing of hair from different individuals, and freezing and crushing solves this problem only to a small extent. This explains the large variation across repeated measurements of samples from the same composite sample. The choice of barbers turned out to be crucial. For the samples to be representative of the ward, it must be confirmed beforehand that only a local customer group is served. The study can definitely not be carried out if the fieldwork is not supported by people who are very familiar with the setting and are trusted in the communities, so very close collaboration of lay staff and research staff is necessary, with the research staff maintaining permanent quality control and oversight.

The main strengths of this study include the first direct human exposure measurement in the area concerned, the large sample, and the protocol that allowed sampling of also the most deprived communities. Weaknesses are that the selection of barbers did perhaps mask some of the difference in exposure levels we had expected to find between the HE and LE wards. Also, major variability was found in measured samples coming from the same composite sample. This suggests that individual variation may be larger than group-level variation, but this hypothesis needs to be addressed with better exposure modelling and a respective measurement validation study. Based on this, it remains an objective to develop an exposure prediction model based on geographical location of residence, taking into account proximity to tailings dams, dust pollution, wind directions, erosion levels, water distribution and consumption, use of local food, and other potential exposure routes (including occupational) into account. The experience of this pilot study shows that validation of predictions in the form of exposure maps would be essential.

## Conclusions

In conclusion, U concentrations measured in the hair of the resident population of this South African gold mining area indicate elevated U levels that merit research on possible adverse health consequences. Further methodological work is needed on whether the exposure likelihood can be better modelled taking into account measured environmental levels, distance to mine tailings, wind directions, water flow, consumption of local water and food, and how common personal habits such as geophagia are. Such prediction models would need to be validated with a new series of individual (rather than composite) human measurements, with a choice of barbers serving fewer commuting customers while serving the most highly exposed indigent population. If it is explained well to the barbers and their customers, collecting hair from barbers appears to be an acceptable, efficient, and non-invasive sampling method, enabling access to people in resource-restricted communities where other methods fail. However, collecting composite samples as a means of minimizing analytical costs is discouraged, due to difficulties in their laboratory analyses.

## Supporting information

S1 Fig Location of sampling areas and barbers in relation to tailings deposits marking the locations of gold mines. (DOCX)

S1 Table Uranium (U) concentrations in hair reported in the literature. (DOCX)
