# Supplementary material for: Human exposure to uranium in South African gold mining areas using barber-based hair sampling
Source: PLoS One. 2019 Jun 27;14(6):e0219059. doi: 10.1371/journal.pone.0219059 (PMC6597193; doi:10.1371/journal.pone.0219059)
Supplement: S1 Fig — (DOCX) [file pone.0219059.s001.docx]

S1 Fig. Location of sampling areas and barbers in relation to tailings deposits marking the location of gold mines
