# Supplementary material for: Human exposure to uranium in South African gold mining areas using barber-based hair sampling
Source: PLoS One. 2019 Jun 27;14(6):e0219059. doi: 10.1371/journal.pone.0219059 (PMC6597193; doi:10.1371/journal.pone.0219059)
Supplement: S1 Table — (DOCX) [file pone.0219059.s002.docx]

**S1Table. Uranium (U) concentrations in hair reported in the literature**

| **Reference**  **(Ref-Figure 2)** | **Study area** | **Study population**  (*Exposure type* and scenario) | **Control/ unexposed**  **U** [µg/kg]  **mean** (min-max), ***median*** | **Exposed**  **U** [µg/kg]  **mean** (min-max), ***median*** |
| --- | --- | --- | --- | --- |
| [40]  **(uw-1)** | Northern Greece | 10 x monks in monastery  *Environmental:* U in drinking water (2.4 µg/l) |  | (12-170)  (n=10) |
| [13]  **(uc-2)** | Iraq,  Fallujah | 26 x adults  *Environmental:* DU ammunition war zone, parents of children born 2009-2010 with major congenital anomalies in Fallujah General Hospital |  | 1^st^ round (all**): 160** (20-400), (n=26)  fathers: **110**  mothers: **180**  2^nd^ round (all): **260** |
| [32]  **(uw-2)** | Northern Sweden | 114 x residents  *Environmental:* high natural U in drinking water |  | **57** (6-436), ***36***  (n=114) |
| [23]  **(ue-1)** | Southern Israel | 99 x volunteers  *Non-exposed* | **62** (10-180), ***50***  (n=99) |  |
| [26]  **(uw-2)** | Brazil,  Sao Paulo | 22 x urban dwellers  *Non-exposed* | **15** (2-50)  (n=22) |  |
| [22]  **(ue-3)**  **(uc-3)**  **(up-3)** | Slovenia,  Lubljana | 40 x exposed and non-exposed adults  *Occupational:* workers at Zirovski Vrh U-mine | All: **14** (3-33)  (n=18), friends and family of authors not working at U-mine  *Males:* **9.5** (3.8-13.5), ***11.5***  (n=7)  Females: **16.1** (7.6-33), ***11.7***  (n=11) | **6225** (40-54500), ***248***  (n=22), all U-mine workers  **102** (40-228), ***47***  (n=3), store + maintenance  **187** (34-484), ***149***  (n=7), U-miners  **947** (151-3610), ***508***  (n=7), U-mill plant  **25740** (5000-54500), ***19900,*** (n=5) yellow cake production |
| [21]  **(ue-9)**  **(ue-10)** | Japan,  Tokyo | 148 x people, 202 x hair samples, 5 x age groups, Tokyo metropolitan area  *Non-exposed* | Males: **38** (5-390)  (n=67)  Females: **51** (8-1280)  (n=81) |  |
| [24]  **(uw-3)** | southern Finland | 852 x adults, 18-66 years old  *Environmental:* high natural U in drinking water, max U in private wells in granite of S-Finland) |  | **216** (0.5-140,000)  (n=852) |
| [38]  **(uw-1)** | South Korea | 18 x workers nuclear laboratory  *Occupational:* workers related to U experiments | **15.5** (<1-39)  (n=10) office/ lab workers not dealing with U directly, | **> 1000**  high dense ‘hot spots’ along single hair (n=8) |
| [25]  **(ue-5)**  **(uw-4)** | Finland | n = 136, 66 x males, 70 x females  134 x water samples,  *Environmental:* elevated U-levels in drinking water: **126** (0.02-1600), ***20*** µg/l) ‘ | **143**  *(n*=68), low-intake sub-group (<10 µg/d) also contained 12 x people who previously used high U water | **8700** (6.5–250,000) ***730*** (n=136) |
| [42] cited in [41] | Balkan |  |  | (0.9-449) |
| Cited in [41] | Brazil |  |  | **150** |
| Cited in [41] | France | *Non-exposed* | (2-30) |  |
| [41]  **(ue-7)** | southern Serbia,  Niski Banja | 26 x adults (40-87 years old), 7 x males (all smokers), 19 x females (1 x smoker), tap water users  *Environmental*: residents of Radium spa town | **14** (0.25-77) ***9.7***  (n=26) |  |
| Cited in [41] |  | 222 x |  | **220**  (n=222) |
| Cited in [41] | Syria | *Occupational*: phosphate miners |  | (965-1838) |
| Cited in [41] |  | 35 x samples  *Occupational* |  | (250-10400)  (n=35) |
| Cited in [41]  **(uc-1)** | Iraq | 250 x samples |  | **220** (<60-2300)  (n=250) |
| [39]  **(ue-8)** | Japan, Tsukuba | 13 x smokers and non-smokers, Tsukuba  *Non-exposed* | (0.4-5.7) ***1.2***  (n=13) |  |
| [29]  **(ue-11)**  **(up-2)** | Namibia | 9 x workers and nearby residents of an U-mine and 5 x controls (n=14),  *Occupational:* U-miners  *Environmental:* town near U-mine | **34** (15-68) ***28***  (n=5), population not working in a uranium mine and not living close to it | **8401** (54-25008) ***142***  (n=3), population working in a uranium mine and living close to the mine  **582** (63-1083) ***620***  (n=6), population not working in a mine but living close to one |
| [30] | South Africa/ France | 8 x black residents, 1 x control  *Environmental:* living near gold mining tailings in the West Rand goldfield (Snakepark, Tudor Shaft) | 15  (n=1), adult white male living in Paris | **33** (13-70) ***30***  (n=8), 7 Black adults (4 male and 3 female) and one 10-months-old baby |
| Cited in [41]  **(uc-5)** | USA | *Occupational:* Sandia National Laboratory |  | **290** (20-1300) |
| **All** | 15 x countries | 22 x studies, total n > 2048 | **9.5-62*** (0.25-1280) ***1.2-50***  12 x studies, total n>329, from 10 x countries  *Karpas et al. (2005) left out due to elevated exposure (i.e. no true controls) | **33-25740** (20*-250,00) ***30-19900***  16 x studies, total n>1442  *Karpas et al. (2005) left out as it contains non-exposed subjects |
